# Taxonomic description of *Micromonospora reichwaldensis* sp. nov. and its biosynthetic and plant growth-promoting potential

**DOI:** 10.1128/spectrum.02129-24

**Published:** 2025-03-03

**Authors:** Imen Nouioui, Alina Zimmermann, Juan Pablo Gomez Escribano, Marlen Jando, Gabriele Pötter, Meina Neumann-Schaal, Yvonne Mast

**Affiliations:** 1 Leibniz-Institute DSMZ–German Collection of Microorganisms and Cell Cultures, Braunschweig, Germany; 2 Braunschweig Integrated Centre of Systems Biology (BRICS), Braunschweig, Germany; 3 Technische Universität Braunschweig, Institut für Mikrobiologie, Braunschweig, Germany; University of the Philippines Los Baños, Laguna, Philippines

**Keywords:** polyphasic taxonomy, bioactive compounds, plant growth-promoting

## Abstract

*Micromonospora* strains proved to be a model organism for drug discovery and plant growth promotion (PGP). Strain DSM 115977 ^T^ was subjected to polyphasic taxonomic analysis and genome mining for biosynthetic gene clusters and PGP-associated genes in order to determine its taxonomic rank and assess its biosynthetic potential. The strain was found to form a novel species within the evolutionary radiation of the genus *Micromonospora*. The strain contained glucose, mannose, xylose, and ribose as whole-cell sugars and the isomer DL-diaminopimelic acid in its peptidoglycan. Strain DSM 115977^T^ had *iso*-C_15:0_, *iso*-C_16:0_, C_17:1_*cis* 9, C_17:0_, *iso*-C_17:0_, and 10-methyl*-*C_17:0_ as fatty acid profile (>5%) and MK10-H_4_ and MK10-H_6_ as the predominant menaquinones (>10%). The polar lipid profile consisted of diphosphatidylglycerol, phosphatidylethanolamine, phosphatidylinositol, glycophosphatidylinositol, glycophospholipids, phosphoaminolipid, unidentified lipids, and phospholipids. The genome of the strain had a size of 7.0 Mbp with a DNA G + C content of 73.4%. It formed a well-supported sub-clade with its close phylogenomic neighbor, *Micromonospora echinofusca* DSM 43913^T^ (98.7%). Digital DNA–DNA hybridization and average nucleotide identity derived from sequence comparisons between the strain and its close phylogenomic neighbors were below the thresholds of 70 and 95–96% for prokaryotic species demarcation, respectively. Based on these findings, strain DSM 115977^T^ (Asg4^T^ = KCTC 59188^T^) merits to be considered as the type strain of a new species for which the name *Micromonospora reichwaldensis* sp. nov. is proposed. Genome mining for biosynthetic gene clusters encoding specialized secondary metabolites highlighted its ability to produce potentially novel therapeutic compounds. The strain is rich in plant growth-promoting genes whose predicted products directly and indirectly affect the development and immune system of the plant.

**IMPORTANCE:**

In view of the significant pharmaceutical, biotechnological, and ecological potentials of micromonosporae, it is particularly interesting to enhance the genetic diversity of this genus by focusing on the isolation of novel strain from underexplored habitats, with the promise that novel bacteria will lead to new chemical entities. In this report, modern polyphasic taxonomic study confirmed the assignment of strain DSM 115977^T^ to a novel species for which the name *Micromonospora reichwaldensis* sp. nov. is proposed. The strain harbors in its genomic sequence several biosynthetic gene clusters for secondary metabolites and genes associated with plant growth-promoting features. The results of this study provide a very useful basis for launching more in-depth research into agriculture and/or drug discovery.

## INTRODUCTION

The genus *Micromonospora* (1) emended by Gao et al. (2) and Nouioui et al. (3) of the family *Micromonosporaceae* (4) and order *Micromonosporales* (5) contains more than 125 validly published species names (https://lpsn.dsmz.de/), with *Micromonospora chalcea* (6) as the type species. Micromonosporae are characterized by the presence of a branched substrate mycelium with non-motile spores and usually the absence of aerial mycelium, which is short and sterile when it occurs in some strains (7). These microorganisms are chemoorganotrophic, aerobic to microaerophilic with *meso*-diaminopimelic acid, and/or 3-OH-diaminopimelic acid in their cell wall peptidoglycan. Whole-cell hydrolysates are rich in xylose and arabinose, which are diagnostic sugars, whereby galactose, glucose, mannose, rhamnose, and ribose may also be present (7, 8). The polar lipid profile includes phosphatidylethanolamine (PE), phosphatidylinositol (PI), and phosphatidylinositolmannosides, with PE as the diagnostic phospholipid. The pattern of fatty acids and menaquinones is complex (3, 8, 9). This taxon has a genome size ranging between 6 and 8 Mbp with a G + C content of 65–75 mol% (3, 8, 9). Micromonosporae strains form a monophyletic genus structured into four well-supported clusters that can be differentiated based on biochemical, enzymatic, and chemotaxonomic features (7, 10). This group of microorganisms has been isolated from different environmental samples, such as soil, rhizosphere (11, 12), nitrogen-fixing root nodules (8, 13), and plants (14, 15), as well as from extreme hyper-arid habitats, such as the Atacama Desert (9).

The integration of genome-scale methods into polyphasic taxonomy has led to significant improvements in the systematics of prokaryotes and a better understanding of the evolutionary process and biotechnological potential (16–18). Comparative genomic approaches based on bioinformatics tools, such as average nucleotide identity (ANI), average amino acid identity (95–96% cut-off values), and digital DNA–DNA hybridization (dDDH) (70% cut-off value) (19–21), have resolved the taxonomic status of several complex and heterogeneous actinobacterial taxa. Modern prokaryotic systematics has clarified the relationships within and between taxa, including the genus *Micromonospora* (3, 8, 22).

Micromonosporae strains have genomes rich in biosynthetic gene clusters (BGCs) associated with specialized metabolites with antibiotic, anti-cancer, or anti-viral activities, some of which are genus-specific substances (8). This is in line with the vision of this actinobacterial genus as a model organism for drug discovery (23). Several compounds from different antibiotic families, for example, aminoglycosides, were extracted from micromonosporae, such as gentamicin, sisomicin, verdamicin, antibiotics G-52, G-418, JI-20, and 460, fortimicins, neomycin, and sagamicin (24). Furthermore, micromonosporae strains from different habitats have multiple genes associated with plant growth promotion (PGP); features that have been confirmed *in vitro* for several *Micromonospora* species, such as *Micromonospora lupini* and *Micromonospora saelicesensis* (8, 25, 26).

Given the significant pharmaceutical, biotechnological, and ecological potentials of micromonosporae, it is particularly interesting to enhance the genetic diversity of this genus, focusing on underexplored habitats, with the promise that novel bacterial strain will lead to novel chemical entities (27).

In the present study, strain DSM 115977^T^ derived from brown coal ash in Germany was subjected to polyphasic taxonomic analysis and genome mining for biosynthetic gene clusters and PGP-associated genes to determine its taxonomic rank and assess its biosynthetic potential. Phenotypic and genomic data confirmed the assignment of the strain to a novel species for which the name *Micromonospora reichwaldensis* sp. nov. is proposed.

## MATERIALS AND METHODS

### Origin, isolation, and maintenance of strain DSM 115977^T^

Strain DSM 115977^T^ was isolated from ash of a brown coal mine in Reichwalde, Germany. The isolation of the strain was carried out as follows: 0.5 g of ash was sprinkled directly onto a DSMZ 65 agar medium containing cycloheximide and nalidixic acid (each at 25 µg/mL) and incubated at 28°C for 14 days (28). A colony of the strain was streaked on DSMZ 65 agar plate and incubated at 28°C for 14 days. The purity of the culture was checked using a light microscope and then deposited at the DSMZ open culture collection and Korean collection for type culture (KCTC) under accession numbers DSM 115977^T^ and KCTC 59188^T^, respectively. The closest phylogenomic neighbor of the strain, *Micromonospora echinofusca* DSM 43913^T^, was obtained from the DSMZ culture collection and is available in the online catalogue (https://www.dsmz.de/collection/catalogue), where all information related to the history, origin, and growth properties of the strain are listed. Phenotypic tests and molecular identification, as well as genome sequencing, were carried out on wet biomass, while chemotaxonomic analyses, except for fatty acids, were performed on freeze-dried cells.

### Molecular identification and genome sequencing of strain DSM 115977^T^

Biomass from a culture grown on ISP2 (DSMZ 987) liquid medium shaken at 150 rpm and incubated at 28°C for 14 days was subjected to DNA extraction, as described in Carro et al. (8). PCR-mediated amplification of the 16S rRNA gene was performed according to Weisburg et al. (29). The 16S rRNA gene PCR product was sequenced using the Sanger method (30). A nearly complete 16S rRNA gene was obtained (>1400 bp), aligned, and compared with all sequences of *Micromonospora* species available at the EzBioCloud database and validly named using the EzBioCloud sever tool (https://www.ezbiocloud.net/).

DNA extraction for whole-genome sequencing was conducted on wet biomass prepared under the growth conditions described above. Extraction, quantitative and qualitative evaluation of DNA, and genomic DNA libraries were undertaken by the MicrobesNG service (Birmingham, UK; https://microbesng.com/). Sequencing was carried out using the Illumina HiSeq instrument and the 250 bp paired-end protocol of the MicrobesNG service. A quantitative assessment of the genome sequence of strain DSM 115977^T^ was estimated using Benchmarking Universal Single-copy Orthologs and based on the *Actinobacteria* phylum database (31). The RAST-SEED webserver with default options was used for genome sequence annotation (32). The genome sequence of strain DSM 115977^T^ was deposited at DDBJ/ENA/GenBank under accession number JAVRFL000000000.1.

### Phylogenetic and comparative genomic studies

The 16S rRNA gene sequence of strain DSM 115977^T^ was compared with those of validly named species available in the EzBioCloud database (33). The reference strains included in this present study were, therefore, retrieved from the EzBioCloud webserver. Pairwise 16S rRNA gene sequence similarity between strain DSM 115977^T^ and its closest phylogenetic relative was determined after the recommended settings proposed by Meier-Kolthoff et al. (34) using the Single-Gene trees online tool available via the Genome to Genome Distance Calculator (GGDC) server (35). Maximum likelihood (ML) and maximum parsimony (MP) phylogenies based on the 16S rRNA gene sequences were inferred using the DSMZ phylogenomic pipeline adapted to single genes, which is available at the GGDC platform (https://ggdc.dsmz.de/home.php) (35–37). MUSCLE program was used for a multiple sequence alignment (38). RAxML (39) and TNT (40) were used for ML and MP trees, respectively. Fast bootstrapping, in combination with the autoMRE bootstopping criterion (41), and a post-search for the best tree were used for ML, while 1,000 bootstrapping, in conjunction with branch permutation by tree bisection and reconnection, and 10 replicates of random sequence additions were used for the MP tree. The *Χ*² test implied in PAUP* was applied to check the sequences for compositional bias (42).

Genome-based taxonomy was inferred via a high-throughput online platform, Type (Strain) Genome Server (TYGS), available at https://tygs.dsmz.de/ (35). The genome sequence of *Asanoa ferruginea* DSM 44099^T^ was used as an outgroup.

Pairwise dDDH hybridization values between the genomic sequence of the isolate and its closest phylogenomic relatives were estimated using TYGS. dDDH values were based on the recommended formula *d_4_*, which reflects the proportion of sequence identity in homologous parts of the underlying genomes and is independent of the genome length (36). The ANI between the genomic sequence of the isolate DSM 115977^T^ and its nearest phylogenomic neighbors was calculated using the ANI calculator software available in the EzbioCloud server (https://www.ezbiocloud.net/tools/ani) (43).

### Chemotaxonomic characterization of strain DSM 115977^T^

Freeze-dried cells of strain DSM 115977^T^ were obtained from a culture prepared on an ISP2 (DSMZ 987) liquid medium shaken at 150 rpm and incubated at 28°C for 14 days. The harvested biomass was washed three times with a sterile saline solution (0.8% NaCl) and then lyophilized. Standard chromatographic procedures were applied to analyze whole-cell sugars (44), diaminopimelic acid isomers of the peptidoglycan (45), and polar lipids (46). The isoprenoid quinone profile was determined using HPLC coupled to a diode array detection and high-resolution spectrometer (47). Wet biomass from the 3-day old culture on medium DSMZ 553SCH of strains DSM 115977^T^ and DSM 43913^T^ was subjected to cellular fatty acid extraction using standard microbial identification system protocols (MIDI Inc.; version 6.1) (48). The identity of the fatty acid peaks was confirmed by gas chromatography–mass spectrometry, as described previously (49).

### Growth properties and biochemical and enzymatic features of strain DSM 115977^T^

The growth properties of the strain were evaluated after assessing its ability to grow on different media and over a wide range of temperatures and pH. Homogeneous bacterial suspension in a 0.5 McFarland interval was used to inoculate ISP1 (International *Streptomyces* Project; DSMZ 1764), ISP2 (DSMZ 987), ISP3 (DSMZ 84), ISP4 (DSMZ 252), ISP5 (DSMZ 993, ISP6 (DSMZ 1269), ISP7 (DSMZ 1619), GYM (DSMZ 65), N-Z-amine agar (DSMZ 554), Nutrient (DSMZ 1), and Czapek’s (DSMZ 83) agar media, followed by incubation at 28°C for 14 days. The growth of the strain was also evaluated in the presence of a wide range of pH (5.0, 5.5, 6.0, 6.5, 7.0, 7.5, 8.0, 8.5, 9.0), temperatures (4, 10, 16, 20, 25, 28, 35, 37, 42, 45°C), and different concentrations of NaCl (0, 2.5, 5, 7.5, 10% w/v) on DSMZ 65 agar plates. The following buffer systems were used to adjust: pH < 6, 0.1 M citric acid/0.1 M sodium citrate; pH = 8, 0.1 M KH_2_PO_4_/0.1 M NaOH; and pH = 9, 0.1 M NaHCO_3_/0.1 M Na_2_CO_3_. The color of the substrate mycelium of the strain was recorded according to RAL color charts (50). The biochemical and enzymatic profiles of the strain and their closest phylogenomic neighbor, *M. echinofusca* DSM 43913^T^, were identified using API 20NE and API ZYM strips (bioMérieux) following the manufacturer’s instructions.

### *In vitro* and *in silico* screening for specialized secondary metabolites

#### Genome mining for secondary metabolite BGCs

The genome sequences of DSM 115977^T^ and its close phylogenomic relative, *M. echinofusca* DSM 43913^T^, were screened for secondary metabolite BGCs using the antiSMASH webtool version 7.0 with default parameters (https://antismash.secondarymetabolites.org) (51).

#### Antimicrobial bioassay

The crude extract of the strain was prepared from a 10-day-old culture (50 mL) grown on NL800 and R5 liquid media and shaken at 180 rpm at 28°C. Extraction was performed using ethyl acetate, as described by Nouioui et al. (52). The reference strains used in this study were the multiple antibiotic-resistant strain *Staphylococcus aureus* DSM 18827 and *Enterococcus faecium* DSM 20477^T^ as the Gram-positive bacteria*, Escherichia coli ΔtolC* JW5503-1 and *Proteus vulgaris* DSM 2140 as the Gram-negative bacteria, and *Candida albicans* DSM 1386 as the pathogenic yeast representative. Details on the growth conditions, origin, and history of these strains are publicly available in the DSMZ online catalogue (https://www.dsmz.de/collection/catalogue). *E. coli ΔtolC* JW5503-1 was obtained from the *E. coli* Genetic Stock Center, Yale (collection number: 11430). Antimicrobial bioassays were performed using a volume of 30 µL methanolic crude extract, as described in Nouioui et al. (52).

#### Bioinformatic analysis for the detection of plant growth-promoting features

The genome of the strains DSM 115977^T^ and DSM 43913^T^ was screened for genes known to directly or indirectly promote plant growth using the PGPT-Pred tool, which is available in a comprehensive platform, called PLaBAse (https://plabase.cs.uni-tuebingen.de/pb/plabase.php) (53–55). Blast and blastp + hmmer approach against the PGPT ontology were used. The results presented in this study were restricted to krona strict mode blastp + hmmer.

## RESULTS AND DISCUSSION

### Taxonomic novelty and distinctive genomic features of strain DSM 115977^T^

The 16S rRNA gene is known as the gold standard molecular marker for phylogenetic studies (56). The 16S rRNA gene sequence similarity values between strain DSM 115977^T^ and the 37 top-hit type strains of *Micromonospora* species validly named ranged from 98.7 to 99.7%, with *Micromonospora phytophila* SG15^T^ as the closest reference strain (Table S1). These results were based on 1,508 characters, 60 of which were variable, and 48 of which were parsimony-informative. In the maximum-likelihood 16S rRNA gene-based tree (Fig. S1), strain DSM 115977^T^ and *M. phytophila* SG15^T^ together formed a poorly supported sub-group loosely associated with a cluster comprising several type strains, including *M. echinofusca* (98.7%). The low bootstrap value of the phylogenetic tree clearly showed that the resolution of the 16S rRNA gene is insufficient to separate closely related species. Therefore, a whole genome-based phylogeny was established to determine the phylogenetic position of the strain within the evolutionary radiation of the genus *Micromonospora*. Comparative genomic analysis was performed between the strain and its close phylogenomic *Micromonospora* species validly named using dDDH and ANI approaches.

**Fig 1:**
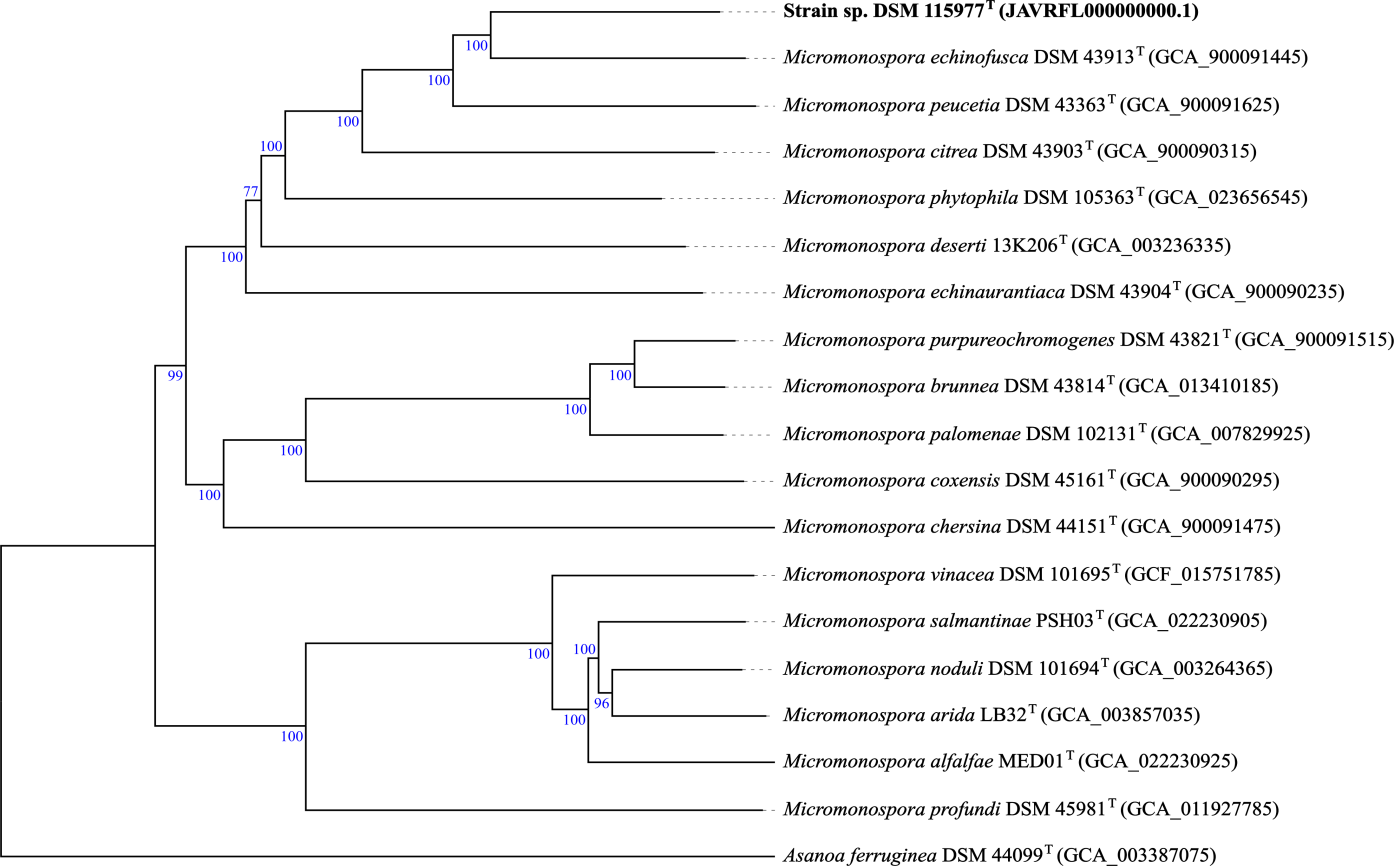
Genome-based phylogeny of strain DSM 115977^T^ and its close phylogenomic relatives inferred by the TYGS web server. The tree was inferred with FastME from the GBDP distances calculated from genome sequences. The branch lengths are scaled in terms of the GBDP distance formula *d_5_*.

The authenticity of strain DSM 115977^T^ was confirmed by a comparison of the 16S rRNA gene sequence obtained from the PCR reaction with that extracted from the whole-genome sequence. The phylogenomic tree revealed that strain DSM 115977^T^ constituted a well-supported sub-clade, with *M. echinofusca* DSM 43913^T^ within the cluster harboring *Micromonospora peucetia* DSM 43363^T^, *Micromonospora citrae* DSM 43903^T^, *M. phytophila* DSM 105363^T^, *Micromonospora deserti* 13K206^T^, and *Micromonospora echinaurantiaca* DSM 43904^T^ (Fig. 1). The genomic sequence of strain DSM 115977^T^ and those of its close phylogenomic neighbors showed dDDH and ANI between 26.4 and 50.2% and 92.8 and 82.8%, values well below the threshold of 70 and 95–96% for prokaryotic species demarcation (19–21, 57) (Table 1), respectively. Consequently, strain DSM 115977^T^ merits to be considered as the type strain of a novel species, for which the name *Micromonospora reichwaldensis* sp. nov. is proposed.

**TABLE 1:** dDDH and ANI between the genome sequence of strain DSM 115977^T^ and its close phylogenomic relatives

Subject strain	Genome accession	dDDH (%)	ANI	G + C content difference (%)
*Micromonospora echinofusca* DSM 43913^T^	GCA_900091445	50.2	92.8	0.1
*Micromonospora peucetia* DSM 43363 ^T^	GCA_900091625	45.8	91.7	1.1
*Micromonospora citrea* DSM 43903 ^T^	GCA_900090315	39.0	89.5	0.3
*Micromonospora phytophila* DSM 105363 ^T^	GCA_023656545	35.2	87.8	1.3
*Micromonospora deserti* 13K206 ^T^	GCA_003236335	32.7	86.8	1.0
*Micromonospora echinaurantiaca* DSM 43904 ^T^	GCA_900090235	31.3	86.1	0.2
*Micromonospora brunnea* DSM 43814^T^	GCF_013410185	28.1	84.7	0.4
*Micromonospora palomenae* DSM 102131^T^	GCA_007829925	28.0	84.6	0.4
*Micromonospora purpureochromogenes* DSM 43821^T^	GCA_900091515	27.8	84.7	0.4
*Micromonospora coxensis* DSM 45161^T^	GCA_900090295	27.7	84.3	0.1
*Micromonospora halophytica* DSM 43171 ^T^	GCA_900090245	27.4	84.1	0.5
*Micromonospora chersina* DSM 44151^T^	GCA_900091475	27.2	83.7	0.1
*Micromonospora harpali* NEAU JC6^T^	GCA_040429615	27.2	83.5	0.4
*Micromonospora okii* TP-A0468^T^	GCA_020884795	27.2	84.0	0.4
*Micromonospora haikouensis* DSM 45626^T^	GCA_900091595	27.2	83.7	0.3
*Micromonospora chalcea* DSM 43026 ^T^	GCA_002926165	27.0	83.3	0.6
*Micromonospora arida* LB32^T^	GCA_003857035	26.9	83.5	2.4
*Micromonospora noduli* GUI43^T^	GCA_003264365	26.9	83.3	2.3
*Micromonospora vinacea* DSM 101695 ^T^	GCF_015751785	26.8	83.4	2.3
*Micromonospora salmantinae* PSH03 ^T^	GCA_022230905	26.7	83.3	2.4
*Micromonospora alfalfae* MED01 ^T^	GCA_022230925	26.6	83.1	2.6
*Micromonospora profundi* DSM 45981^T^	GCA_011927785	26.4	82.9	2.6
*'Micromonospora zhangzhouensis'* HM134^T^	GCA_007833915	26.3	83.1	0.5
*Micromonospora matsumotoense* DSM 44100^T^	GCA_900091525	26.3	82.9	1.1
*Micromonospora rifamycinica* AM105^T^	GCA_001542325	26.3	82.9	0.4
*Micromonospora wenchangensis* CCTCC AA 2012002^T^	GCA_002210435	26.2	83.0	0.4
*Micromonospora humidisoli* MMS20-R2-29^T^	GCA_016900955	26.0	82.8	0.5
*Micromonospora rifamycinica* DSM 44983^T^	GCA_900090265	26.0	82.8	0.4
*Micromonospora antibiotica* MMS20-R2-23^T^	GCA_017599305	26.0	82.6	0.9
*Micromonospora humida* MMS20-R1-14^T^	GCF_016901255	25.9	82.9	0.5
*Asanoa ferruginea* DSM 44099^T^	GCA_003387075	21.5	^*a*^-	2.6

^*a*^ -, not applicable.

In order to investigate the genetic similarity and identify the functional differences between the strain and its close relative, *M. echinofusca*, the two genome sequences were analyzed in detail. The draft genome sequence of strain DSM 115977^T^ was obtained by Illumina sequencing and found to have 99.3% completeness. Strain DSM 115977^T^ and its close neighbor, *M. echinofusca* DSM 43913^T^, have a genome size of 7.05 and 7.00 Mbp, G + C contents of 73.4 and 73.3%, total RNA numbers of 56 and 66, and 6444 and 6314 coding sequences, respectively (Table 2). The genomic features of strains DSM 115977^T^ and DSM 43913^T^ were within the known range for the genus *Micromonospora* (genome size 6–8 Mbp, average 7  ±  0.4, and G + C content of 71.1–73.8 mol [8]). Strains DSM 115977^T^ and DSM 43913^T^ exhibited 295 and 289 subsystems, respectively, based on the RAST annotation and divided into ~23–24 categories, as detailed in Table 3. Both strains seemed to have acquired some resistance to heavy metal, such as cobalt–zinc–cadmium and copper, as well as against antibiotics, such as β-lactamase and fluoroquinolones, due to the occurrence of respective resistance genes. In addition, the strains possessed gene potentially conferring the enzymatic ability to reduce mercury.

**TABLE 2:** Genomic features of strain DSM 115977^T^ and its close phylogenomic neighbor, *M. echinofusca* DSM 43913^T^

Genomic features	Strain DSM 115977^T^	*Micromonospora echinofusca* DSM 43,913^T^
Genome Size (Mb)	7.0	7.0
Contigs	153	1
N50 (bp)	159436	1
GC (%)	73.4	73.3
Number of RNAs	56	66
Number of coding sequences	6444	6314
Genome completeness (%)	99	^*a*^-
Contamination (%)	1	-
GenBank accession numbers	JAVRFL000000000.1	NZ_LT607733.1

^*a*^ -, not applicable.

**TABLE 3:** The subsystem categories of strain DSM 115977 ^T^ and its close phylogenomic relative, DSM 43913^T^, ranked in descending order of the numbers of CDS

	DSM 115977^T^	DSM 43,913^T^
Amino acid and derivatives	305	277
Carbohydrates	281	241
Protein metabolism	210	215
Cofactors, vitamins, prosthetic groups, and pigments	169	157
Fatty acids, lipids, and isoprenoids	126	110
DNA metabolism	106	103
Respiration	92	98
Nucleosides and nucleotides	77	77
Metabolism of aromatic compounds	52	43
Membrane transport	54	46
RNA metabolism	51	49
Stress response	51	38
Cell wall and capsule	40	48
Virulence, disease, and defense	40	37
Antibiotic and toxic compounds	28	25
invasion and intracellular resistance	12	12
Iron acquisition and metabolism	27	21
Phosphorus metabolism	22	23
Regulation and cell signaling	22	19
Nitrogen metabolism	21	21
Sulfur metabolism	11	9
Dormancy and sporulation	8	8
Potassium metabolism	7	5
Secondary metabolism	6	2

### Growth and biochemical, enzymatic, and chemotaxonomic properties of strain DSM 115977^T^

Since the phylogenomic and genetic data confirmed the assignment of strain DSM 115977^T^ to a novel species within the genus *Micromonospora*, it is important to provide the growth properties of the proposed type strain and its phenotypic features in comparison with its nearest phylogenomic neighbour.

Strain DSM 115977^T^ showed morphological and phenotypic traits consistent with the genus *Micromonospora* (8). The strain formed an extensively branched orange substrate mycelia on ISP1 (DSMZ 1764), ISP2 (DSMZ 987), ISP6 (DSMZ 1269), ISP7 (DSMZ 1619), GYM (DSMZ 65), N-Z-amine (DSMZ 554), TSA (trypticase soy agar; DSMZ 535), and nutrient agar (DSMZ 1), where a good growth was recorded. Poor to moderate growth of the strain was observed at 17, 20, and 25°C, while good growth was detected at 28, 35, and 37°C on the DSMZ 65 medium after 14 days of incubation. The strain was able to grow well at 42°C, but the optimal temperature for growth was at 28 and 37°C. The strain was able to grow in the presence of up to 2.5% NaCl.

Strain DSM 115977^T^ could be distinguished from its close phylogenetic neighbor, *M. echinofusca* DSM 43913^T^, by its ability to produce α- and ß-galactosidase, ß-glucuronidase, α- and β-glucosidase, and N-acetyl-β-glucosaminidase and by its capability to metabolize potassium nitrate, D-mannitol, and malic acid (Table 4).

**TABLE 4:** Biochemical and enzymatic features that distinguish strain DSM 115977^T^ from its close phylogenomic neighbor, *M. echinofusca* DSM 43913^T*b*^

Phenotypic traits^*a*^	DSM 115977^T*c*^	DSM 43913^T*c*^
α-Galactosidase	+	-
β-Galactosidase	+	-
β-Glucuronidase	+	-
α-Glucosidase	+	-
β-Glucosidase	+	-
N-Acetyl-ß-glucosaminidase	+	-
Biochemical traits		
Potassium nitrate	+	+
D-Glucose	-	+
D-Mannitol	+	-
D-Maltose	-	+
Adipic acid	-	+
Malic acid	+	-
Chemotaxonomic features		
Diaminopimelic acid	DL-DAP	DL-DAP^*b*^
Whole-cell sugar	Glu, Man, Xyl, Rib	Glu, Xyl^*b*^
Polar lipid profile	DPG, PE, PI, GPI, GPL_s_; PAL, PL_1-5_, Ls	PE^*b*^
Quinone profile	MK10-H_4_ (63.1%), MK10-H_6_ (30.6%)	MK9-H_4_^*b*^
Fatty acid profile (>5%)	*iso*-C_15:0_ (23.9%), *iso-*C_16:0_ (18.5%), C_17:1_ *cis* 9 (11.7%), C_17:0_ (7.8%), 10-methyl-C_17:0_ (6.8%), *iso*-C_17:0_ (6.8%)	*iso*-C_16:0_ (22.2%), *iso-*C_15:0_ (22.1%), C_17:1_ *cis* 9 (11.8%), C_17:0_ (7.3%)_,_*iso*-C_17:0_ (5.4%)

^*a*^ Glu, glucose; man, mannose; xyl, xylose; rib, ribose. DPG, diphosphatidylglycerol; PI, phosphatidylinositol; PE, phosphatidylethanolamine; PL_1-4_, phospholipids; GPI, glycophosphatidylinositol; GPL, glycophospholipid; PAL, phosphoaminolipid; PGL_1-2_, phosphoglycolipids. Both strains had positive reactions for alkaline phosphatase, esterase (C4), esterase lipase (C8), lipase (C14), leucine arylamidase, valine arylamidase, cystine arylamidase, trypsin, a-chymotrypsin, acid phosphatase, naphthol-as-bi-phosphohydrolase, esculin, ferric citrate, gelatin (bovine origin), 4-nitrophenyl-ß-D-galactopyranoside, L-arabinose, and potassium gluconate. Both strains had negative reactions for a-mannosidase, α-fucosidase, L-tryptophan, L-arginine, urea, D-mannose, N-acetylglucosamine, capric acid, trisodium citrate, and phenylacetic acid; Positive result; Negative result.

^*b*^ Data taken from Kroppenstedt et al. (58).

^*c*^ (-) negative result; (+) positive result.

Whole-cell hydrolysates of strain DSM 115977^T^ were rich in *meso*-diaminopimelic acid, glucose, mannose, ribose, and xylose, which were in concordance with the diagnostic sugars glucose and xylose of the close phylogenomic neighbor, *M. echinofusca* (58). The major polar lipids of strains DSM 115977^T^ were DPG, PE, and PI. These data were in line with those of *M. echinofusca* (Fig. S2) (58). The predominant menaquinones (>10%) of strain DSM 115977^T^ were MK10-H_4_ (63.1%) and MK10-H_6_ (30.6%), while the type strain of *M. echinofusca* had MK9-H_4_ as the main menaquinone (58). The fatty acid profile (>5%) for strain DSM 115977^T^ consisted of *iso*-C_15:0_, *iso*-C_16:0_, C_17:1_ *cis* 9, C_17:0_, *iso*-C_17:0_, and 10-methyl*-*C_17:0_ in line with those of *M. echinofusca* DSM 43913^T^ (Table 4).

Based on phenotypic, genetic, and genomic data, strain DSM 115977^T^ merits to be considered as a type strain for a novel species for which the name *Micromonospora reichwaldensis* sp. nov. is proposed.

### Antibiotic activity of the studied strains and their secondary metabolite BGCs

Several compounds from different antibiotic families were isolated from *Micromonospora* strains. Gentamicin was first isolated in 1963 from *Micromonospora echinospora* NRRL 2953, *M. echinospora* NRRL 2985, *M. echinospora* subsp. *ferruginea* NRRL 2995, and *M. echinospora* subsp. *pallida* NRRL 2996. Sisomicin, verdamicin, fortimicin, neomycin, G-52, G-418, and JI-20 were derived from *Micromonospora inyoensis* NRRL 3292, *Micromonospora grisea* NRRL 3800, *Micromonospora olivoasterospora* (ATCC 21819, ATCC 31010, and ATCC 31009), *Micromonospora* sp. 69–683, *Micromonospora zionensis* NRRL 5466, *M. echinospora* NRRL 5326, and *Micromonospora purpurea* JI-20, respectively. Sagamicin was initially extracted from *Micromonospora sagamiensis* ATCC 21803 and *Micromonospora* sp. ATCC 21826. As macrolide antibiotics, megalomicins were first recovered from uncharacterized *Micromonospora* strains, while rosaramicin, juvenimicin, erythromycin B, and the antibiotic, M-4365 complex, were first derived from *Micromonospora rosaria* NRRL 3178, *Micromonospora chalcea* ATCC 21561, *Micromonospora* sp. 1225, and *Micromonospora capillata,* respectively. Moreover, *Micromonospora halophytica* NRRL 2998 and *M. halophytica* NRRL 3097 were a source of the ansamycin family of antibiotics, namely, halomicins, while *Micromonospora ellipsospora* 71372 was the first source of rifamycins SV (24). The citreamicins were initially extracted from *Micromonospora citrea* (59, 60). Given the genetic capacity of *Micromonospora* strains to produce bioactive compounds, strain DSM 115977^T^ was subjected to an *in vitro* and *in silico* assessment of its biosynthetic potential. To evaluate the antimicrobial activity of the culture extracts of strain DSM 115977^T^, antibacterial bioassays were carried against a panel of Gram-positive and -negative test bacteria, as well as yeast. Strain DSM 115977^T^ showed antibacterial activities against *E. coli ΔtolC* JW5503-1, *Proteus vulgaris* DSM 2140, and the methicillin-resistant *Staphylococcus aureus* DSM 18827 strain, while no inhibition zone was observed against *Candida albicans* DSM 1386 (Table S2). To match the bioactivity with the genetic biosynthetic potential of the strain, the genome sequence of strain DSM 115977^T^ was analyzed with antiSMASH for the identification of secondary metabolite BGCs. Genome mining of DSM 115977^T^ revealed the presence of 29 BGCs encoding different types of antibiotic classes (e.g., ribosomally synthesized and post-translationally modified peptides, non-ribosomal peptides, thiopeptides, polyketides, or terpenes) (Table S3). All BGCs showed cluster similarity values below 50%, except for three BGCs, which showed similarity to a chitinimide BGC (region 3.1, 58%), a loseolamycin BGC (region 11.1, 60%), and a BGC potentially encoding the morphogenetic peptide SapB (region 19.1, 100%).

The phylogenetically closely related strain DSM 43913^T^ has 26 BGCs encoding for tetrachlorizine, lymphostin, chitinimide, sporolide, SapB, quinolidomicin, hygrocin, ectoine, azicemicin, phosphonoglycan, and loseolamycin. However, here it should be noted that the antiSMAH compound designation has no significance with regard to the substance actually encoded by the BGCs, as the gene cluster similarities are very low. These data should rather give an idea of how similar the biosynthetic performance of the two strains is. From the genome sequences of all *Micromonospora* strains available in the antiSMASH database (*n* = 70 genomes) (51), three BGCs, namely, loseolamycin, phosphonoglycan, and tetrachlorizine, were found to be present in 70, 66, and 63 strains, respectively. However, here it should be noted that there is certainly a specific gene synteny that is abundant in all of these *Micromonospora* strains, but the set of genes most likely does not encode the biosynthesis of those substances since BGC similarity values are rather low. Highest similarity values were found for the loseolamycin BGCs (>60%), which indeed may encode for a compound similar to loseolamycin, a type III polyketide synthase-derived bioactive alkylresorcinol, which has been identified after heterologous expression from *M. endolithica* (61). However, for the detected phosphonoglycan and tetrachlorizine BGCs, only two and three genes each showed similarity to the respective known BGCs, thereby not allowing any statement about the biosynthesis. Another BGC was also found to be very abundant in *Micromonospora* strains, which was an isorenieratene BGC present in 51 (73%) of the 70 strains. This BGC was also identified in the closely related neighbor, *M. echinofusca* DSM 41913^T^.

### Plant growth-promoting features of strain DSM 115977^T^ and its closest phylogenetic neighbor, strain DSM 43913^T^

The ecological potential of *Micromonospora* strains linked to plant growth promotion (PGP) has already been reported in several studies (12). Genome screening of strains DSM 115977^T^ and its close phylogenomic neighbor, DSM 43913^T^, revealed the presence of genes whose products have direct and indirect effects on promoting and protecting plant growth. Both strains possessed PGP genes associated with phytohormone and plant signal production (9%), bioremediation (9%), biofertilization (11%), competitive exclusion (bacterial species entering into competition with other bacterial species for available nutrients and mucosal adhesion sites) (20%), stress control (22%), and plant system colonization (27%). This study provides an insight into the ecological potential of the strains.

Iron (Fe) is one of the crucial micronutrients for most of organisms, including bacteria and plants, and its availability in the soil in insoluble oxidized form (Fe^3+^ oxy-hydroxide) adversely affects photosynthesis and the productivity of plants, especially in those where growth is highly dependent on Fe content, such as quince (*Cydonia oblonga*) (62, 63). Fe^3+^ oxy-hydroxide must be reduced to Fe^2+^ before it can be assimilated by plants, which have (i) the reduction mechanism and (ii) the chelation process in the roots (64, 65). However, these strategies proved insufficient for Fe uptake by plants under iron deficiency conditions (66, 67), which justifies the widespread use of synthetic iron chelating agents in agriculture (68). These fertilizers are able to bind heavy metals, which explains their poor degradation in the soil (69). The saprophytic and symbiotic bacteria that colonize the rhizosphere, along with the endophytic bacteria that thrive inside the plant, have been found to promote the growth and health of the plants. These microorganisms are referred as PGP rhizobacteria (PGPR). The latter exhibit various iron acquisition systems and play an important role in regulating iron uptake in case of soil deficiency (63), in addition to their role in nutrient uptake and reduction of biotic and abiotic stresses (70, 71). Among the known PGPR organisms are *Micrococcus*, *Pseudomonas*, *Agrobacterium*, and *Bacillus* (3, 70). Several *Micromonospora* strains have been shown to promote plant growth, such as *Micromonospora lupini* Lupac 08 and *Micromonospora saelicesensis* GAR05 and PSN13 (8, 25–27).

The genomes of the studied strains comprised gene clusters linked to iron acquisition mechanisms, including hemophores-heme, hemophores-siroheme, iron homeostasis-Fmn|Dmk|Ppl|Ndh|Eet, FoxABCD, and other cytochrome-related protein systems. The genomes of the strains also comprised genes associated with (i) iron uptake regulation (*furB, troR* etc) and (ii) lipoic acid chelators, as well as gene clusters for the iron transport system (*feuABC* and iron III transport) (Table S4). More details on the gene associated with iron metabolism are provided in Table S4.

Phosphorus (P) and potassium (K) are involved in many biological and metabolic processes that are essential for plant growth. Several bacteria have been proven capable of solubilizing inorganic phosphate and potassium and rendering them available to the plant (72). The generation of the assimilable form of P by PGP bacteria is achieved via an enzymatic process or through the production of other molecules, such as siderophores, hydroxyl ions, CO_2_ and organic acids (73–75).

Potassium deficiency is more associated with saline soils, where sodium concentration is high, and, consequently, plants suffer from an imbalance in the cytosolic K^+^/Na^+^ ratio, which is crucial for plant growth (76–78). Only 2% of K is present in the soil in an available form for the plant, while 98% occurs in fixed form (79). Potassium is a macronutrient that is as important as nitrogen (N) for several cereal crops (80, 81). This cation helps plants to take up N and P efficiently, assimilate CO_2_ more effectively, and regulate osmotic balance in the presence of abiotic and biotic stresses (82, 83). It has been shown that K deficiency causes severe deterioration of the plants at various growth stages, leading to a significant reduction in yield quality and quantity (72, 81). PGPR have been demonstrated to produce a number of metabolites that reduce the detrimental impact of abiotic stress on plants by adjusting the ionic transport of potassium and sodium, leading to a balanced Na^+^/K^+^ ratio (84, 85). PGPR synthesize enzymes and exopolysaccharides to support biofilm formation around the root, which limits sodium infiltration (86). Among the microorganisms capable of solubilizing K and P are *Rhizobium*, *Micrococcus*, and *Aspergillus* (87). *Micromonospora* strains have also been shown to be useful for enhancing plant growth and phytoremediation (12). In this context, the genome sequence of the studied strains was found to harbor a multitude of genes associated with phosphate and potassium solubilization and whose products are involved in several organic acids’ biosynthesis, such as acetic, aconitic, butyric, citric, formic, fumaric, keto-gluconate, ketoglutaric, lactic, malic, malonic acid, oxalacetic, propionic, pyruvic, and succinic acids (Table S4). These latter dissolve insoluble phosphate and potassium and make them available to plants.

Environmental contamination by heavy metals is constantly increasing as a result of natural and anthropogenic activities related to mining, the use of chemical pesticides and fertilizers, and industrial waste (88). However, some metals, such as copper, iron, manganese, nickel and zinc, are essential in low concentration for the biological and physiological mechanisms of living organisms, while other metals like arsenic, cadmium, chromium, and mercury are toxic compounds (89). The use of bacteria for bioremediation is an economic and ecological alternative, as they are apt to remediate a contaminated site by absorption or bioaccumulation (90, 91). Apart from PGPR, numerous endophytic bacteria have been found very useful in phytoremediation, as they supply their host with the metabolic energy needed to reduce the phytotoxic effect of heavy metals and/or degrade the heavy metal absorbed by the host plant (92–94). Among these endophytic bacteria, *Micromonospora metallophores* was found to be resistant to toxic compounds and to produce metallophores against various heavy metals (95). Genome analysis indicated that strains DSM 115977^T^ and DSM 43913^T^ have the genetic machinery for metal acquisition and detoxification of heavy metals, such as antimony, arsenic, cadmium, bismuth, chromate, cobalt, copper, nickel, manganese, selenium, tellurium, zinc, etc. (Table S4). Moreover, the strains comprised genes, of which the potential products are involved in the xenobiotic’s biodegradation pathways for several toxic compounds, such as dioxin, atrazine, benzoate, caprolactam, dichloroethane, etc. (Table S4).

PGPR can directly stimulate plant growth by synthesizing phytohormones (96, 97) and metabolizing growth-inhibiting compounds (98). The plant hormonal system can change following microbial interaction (99, 100), which leads to increased biotic and abiotic resistance and regulate the osmotic and antioxidant systems of the plants (101). Among the most well-known phytohormones are auxin (indole-3-acetic acid, IAA), abscisic acid (ABA), gibberellin, and cytokinins, which are known for their crucial role in plant growth. Auxin regulates plant cell elongation and growth, enhances plant immunity, and confers resistance to biotic and abiotic stresses, though it is mainly known for its ability to stimulate root development (100–102). ABA protects the plant against abiotic stress, such as drought and salt, by closing the stomata to reduce water loss and enhance the root elongation when it is in a high concentration (103, 104). Cytokinins induce stomatal opening to boost the photosynthesis and plant growth (103, 105) and confer on the plant certain resistance to abiotic stress, such as drought (106). Gibberellin is involved in regulating different growth stages of the plant from seed germination, stem elongation, flowering, to the development of fruits and seeds (107). It provides the plant with some resistance to both salt and biotic stresses by acting as inter-phytohormonal crosstalk and supporting the symbiotic interaction of plant and bacteria, respectively.

These phytohormones have been recorded in several actinobacterial strains, such as *Frankia*, *Streptomyces,* and *Micromonospora,* in addition to the well-studied PGPR bacteria (e.g., *Rhizobium,* etc.) (8, 108). Genome mining for PGP genes showed that strains DSM 115977^T^ and DSM 43913^T^ have genes of which the potential products are involved in the metabolisms of IAA, ABA, γ-aminobutyric acid (GABA), cytokinin, xanthine, and gibberellins (Table S4). GABA plays a main role in controlling environmental stress responses (109). The genome sequences of the strains harbor genes potentially encoding the biosynthesis of the plant vitamins B12, B1, B2, B5, B6, B9, and C, which are involved in the biosynthesis of cobalamin, thiamine, riboflavin, pantothenic acid, pyridoxine/pyridoxal/pyridoxamine, folate, and ascorbic acid, respectively. Genes related to synthesis of the plant vitamins E, K, and lipoic acid were also detected in the strains.

These findings are in line with the genetic ability of strains DSM 115977^T^ and DSM 43913^T^ to colonize plants, though they were isolated from the ash of brown coal and chukar excrement, respectively (58). A series of genes linked to plant host invasion factors, surface adhesion and attachment mechanisms, and root colonization were found in the genomes of the strains. As other micromonosporae, several genes involved in the metabolism of trehalose were present in the studied strains, with some of them encoding the trehalase enzyme that regulates plant nodule formation (12, 110, 111).

Furthermore, the genetic makeup of the strains contains genes involved in neutralizing osmotic (e.g., ROS scavenging), salinity (e.g., via proline metabolism), acidic (e.g., spermidine), and herbicidal (e.g., organophosphate degradation) stresses. Genes related to heat shock proteins, high and low temperature regulation, and cold-affected biofilm were present in both studied strains. The latter had acidic stress encoding genes related to metabolism of putrescine and oxalate, which induce acid tolerance. Genes associated with induction of systemic resistance to boost the plant immune system were also found in the strains (Table S4).

It is evident from fitness-associated genes that the strains are capable of adapting their metabolism and surviving in a poor and/or competitive environment. This conclusion was drawn after detection of a number of genes connected to the CRISPR-CAS system, adaptative mutation, mobile elements, multidrug resistance, phage defense system, resistance to antimicrobial and toxic compounds (e.g., β-lactam, lanthibiotic, macrolide, etc.), toxin and antitoxin systems, etc. (Table S4). Further details on the bacteria fitness genes are provided in Table S4. Altogether, this discloses the potential plant growth-promoting properties of strain DSM 115977^T^.

### Conclusion

Modern polyphasic taxonomic study confirms the affiliation of strain DSM 115977^T^ to a novel species for which the name *Micromonospora reichwaldensis* sp. nov. is proposed. The strain harbors in its genomic sequence several biosynthetic gene clusters for secondary metabolites and genes associated with plant growth-promoting features which is coherent with the biotechnological and ecological potentials of this taxon (12). The results of this study provide a very useful basis for launching more in-depth research into agriculture and/or drug discovery and call for applying a modern polyphasic taxonomic approach for description of any strains with a potential application (e.g., pharmacy, bioremediation, etc.) and/or with clinical relevance in accordance with the Rules of the Prokaryotic Code of the International Code of Nomenclature of Prokaryotes in order to ensure continuity of research studies by the scientific community.

### Description of *Micromonospora reichwaldensis* sp. nov

*Micromonospora reichwaldensis* (*reich.wald.en’sis*. N.L. fem. adj. *Reichwaldensis,* referring to the Reichwalde village in Germany, from where the brown coal ash was collected)

Gram-stain positive, aerobic actinobacterium with extensively branched substrate mycelium with orange color on ISP1 (DSMZ 1764), ISP2 (DSMZ 987), ISP6 (DSMZ 1269), ISP7 (DSMZ 1619), N-Z-amine (DSMZ 554), and TSA (trypticase soy agar; DSMZ 535) agar media. The strain grows well from 25 to 42°C, optimally at 28 and 37°C and from pH 5.5 to 8.0, optimally at pH 7.0. Optimal growth is observed on ISP1, ISP 6, ISP 7, and N-Z Amine at 28°C.

The strain is oxidase-negative and catalase-positive. Whole-cell sugars consist of glucose, mannose, xylose, and ribose. The peptidoglycan contains DL-DAP. The fatty acid profile (>5%) comprises *iso*-C_15:0_, *iso*-C_16:0_, C_17:1_ *cis* 9, C_17:0_, *iso*-C_17:0_, and 10-methyl*-*C_17:0_. Polar lipid pattern consists of diphosphatidylglycerol, phosphatidylethanolamine, phosphatidylinositol, glycophosphatidylinositol, glycophospholipids, phosphoaminolipid, unidentified lipids, and phospholipids. The predominant menaquinones (>10%) are MK-10 H_4_ and MK-10 H_6_.

The type strain, DSM 115977^T^ (= Asg4^T^ =KCTC 59188^T^), was isolated from brown coal ash collected in Germany. The genome size of strain DSM 115977^T^ is 7.0 Mb, with a DNA G + C content of 73.4%. The GenBank accession number of the assembled draft genome is JAVRFL000000000.1.

## Data Availability

All data generated or analyzed during this study are included in this published article. The accession number of genome data set of strain DSM 115977^T^ used during the current study is available in GenBank under accession number JAVRFL000000000.1.

## SUPPLEMENTAL MATERIAL

Supplemental material spectrum.02129-24-s0001.docx Fig. S1 and S2; Tables S1 to S3.

Table S4 spectrum.02129-24-s0002.xlsx Genes associated with plant growth promotion of strain DSM 115977^T^ and its closest phylogenomic neighbor strain, DSM 43913T.

ASM does not own the copyrights to Supplemental Material that may be linked to, or accessed through, an article. The authors have granted ASM a non-exclusive, world-wide license to publish the Supplemental Material files. Please contact the corresponding author directly for reuse.
