# Supplementary material for: Taxonomic description of Micromonospora reichwaldensis sp. nov. and its biosynthetic and plant growth-promoting potential
Source: Microbiol Spectr. 2025 Mar 3;13(4):e02129-24. doi: 10.1128/spectrum.02129-24 (PMC11960110; doi:10.1128/spectrum.02129-24)
Supplement: Supplemental material — Fig. S1 and S2; Tables S1 to S3. [file spectrum.02129-24-s0001.docx]

**Taxonomic description of *Micromonospora reichwaldensis* sp. nov. and its biosynthetic and plant growth promoting potential**

Imen Nouioui^1^*, Alina Zimmermann^1^, Juan Pablo Gomez Escribano^1^, Marlen Jando^1^, Gabriele Pötter^1^, Meina Neumann-Schaal^1,2^, Yvonne Mast^1,2,3^

^1^Leibniz-Institute DSMZ – German Collection of Microorganisms and Cell Cultures, Inhoffenstraße 7B, 38124 Braunschweig, Germany

^2^Braunschweig Integrated Centre of Systems Biology (BRICS), Rebenring 56, 38106 Braunschweig, Germany

^3^Technische Universität Braunschweig, Institut für Mikrobiologie, Rebenring 56, 38106 Braunschweig, Germany

Corresponding author: Imen Nouioui, imen.nouioui@dsmz.de


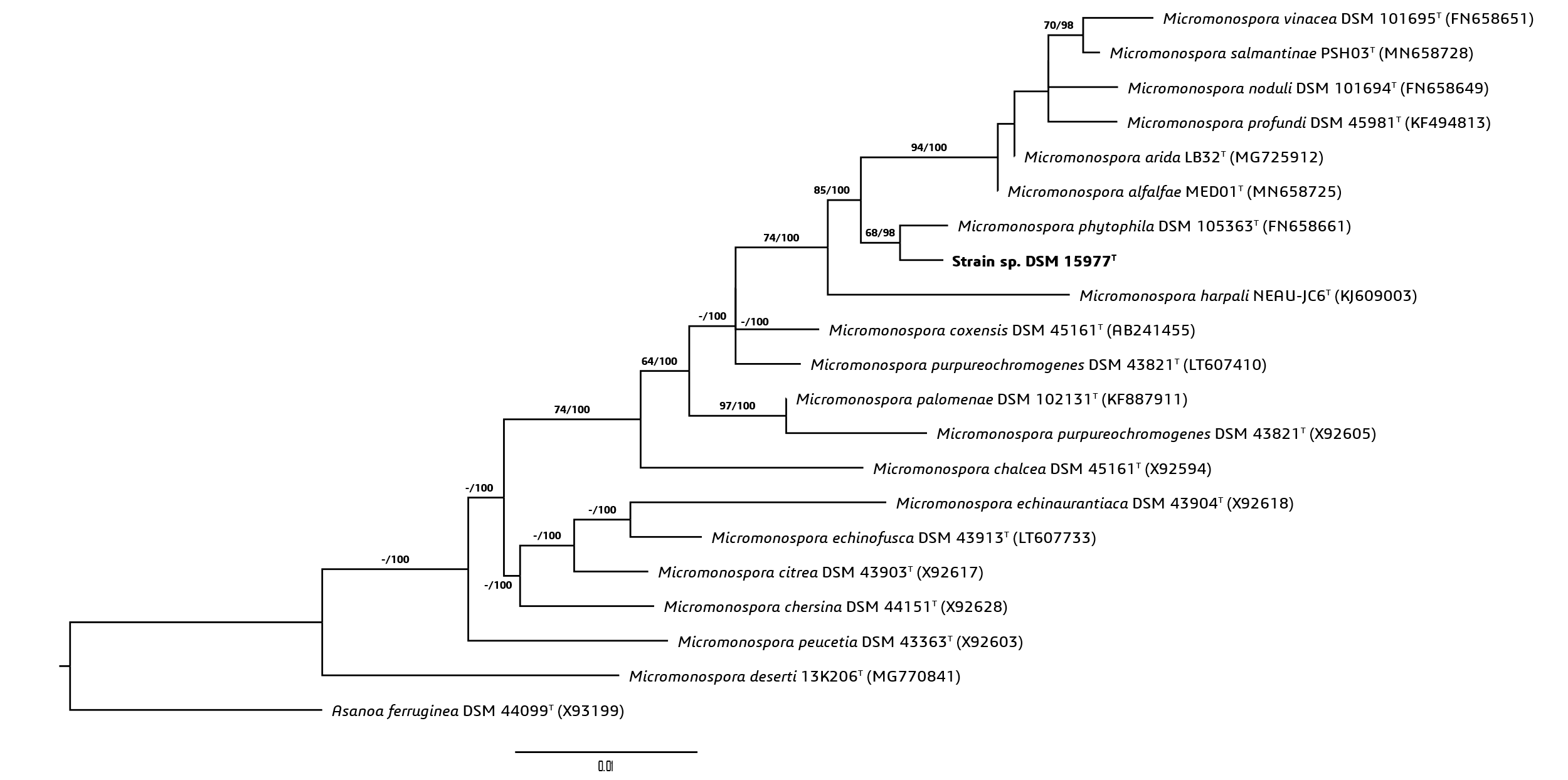


**Figure S1.** ML-phylogenetic tree based on the 16S rRNA gene sequences showing the phylogenetic position of strain DSM 115977^T^ within the radiation of the genus *Micromonospora.* The tree was inferred under the GTR+GAMMA model and rooted by the outgroup sequence of *Asanoa ferruginea*. The branches are scaled in terms of the expected number of substitutions per site. The numbers above the branches are support values when greater than 60% from ML (left) and MP (right) bootstrapping.

**
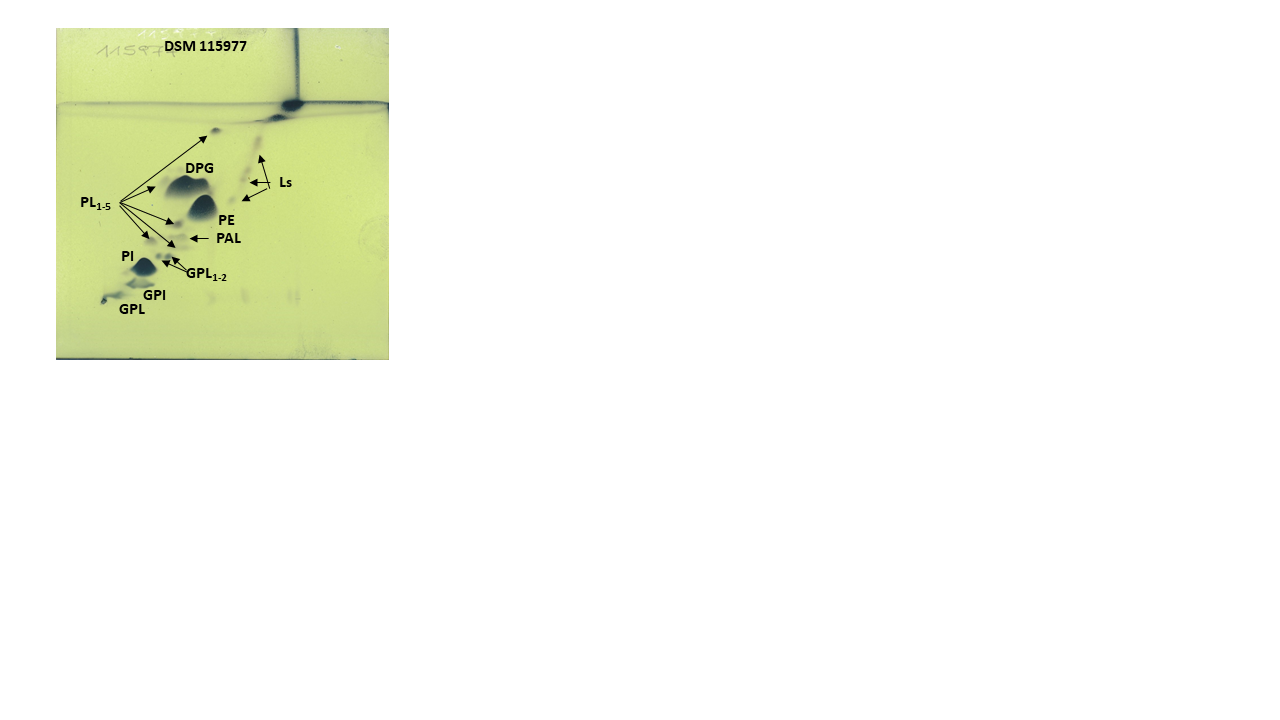
**

**Figure S2.** Two-dimensional TLC plate of polar lipids extracted from the studied strain DSM 115977 stained with molybdatophosphoric acid (SigmaP1518). Key: DPG, diphosphatidylglycerol; PI, phosphatidylinositol, PE, phosphatidylethanolamine, PL_1-5_, phospholipids; Ls, Lipids; GPI glycophosphatidylinositol; GPL glycophospholipids: PAL phosphoaminolipid. Solvent1: chloroform: methanol: distilled water (65:25:4 v/v/v/); solvent 2: chloroform: glacial acetic acid: methanol: distilled water (80:12:15:4 v/v/v).

**Table S1.** 16S rRNA gene sequence similarity between strain DSM 115977^T^ and its close phylogenetic neighbours

| **Query strain** | **Subject strain** | **Accession numbers** | **16S rRNA gene sequence similarity %** |
| --- | --- | --- | --- |
| Strain sp. DSM 115977 | *Micromonospora echinofusca* DSM 43913^T^ | LT607733 | 98.81 |
| Strain sp. DSM 115977 | *Micromonospora peucetia* DSM 43363^T^ | X92603 | 98.93 |
| Strain sp. DSM 115977 | *Micromonospora citrea* DSM 43903^T^ | X92617 | 98.86 |
| Strain sp. DSM 115977 | *Micromonospora phytophila* DSM 105363^T^ | FN658661 | 99.69 |
| Strain sp. DSM 115977 | *Micromonospora deserti* 13K206^T^ | MG770841 | 98.19 |
| Strain sp. DSM 115977 | *Micromonospora echinaurantiaca* DSM 43904^T^ | X92618 | 98.17 |
| Strain sp. DSM 115977 | *Micromonospora palomenae* DSM 102131^T^ | KF887911 | 99.48 |
| Strain sp. DSM 115977 | *Micromonospora purpureochromogenes* DSM 43821^T^ | LT607410 | 99.48 |
| Strain sp. DSM 115977 | *Micromonospora coxensis* DSM 45161^T^ | AB241455 | 99.63 |
| Strain sp. DSM 115977 | *Micromonospora chersina* DSM 44151^T^ | X92628 | 98.93 |
| Strain sp. DSM 115977 | *Micromonospora harpali* NEAU-JC6 ^T^ | KJ609003 | 98.96 |
| Strain sp. DSM 115977 | *Micromonospora chalcea* DSM 45161^T^ | X92594 | 98.86 |
| Strain sp. DSM 115977 | *Micromonospora noduli* DSM 101694^T^ | FN658649 | 99.38 |
| Strain sp. DSM 115977 | *Micromonospora arida* LB32^T^ | MG725912 | 99.39 |
| Strain sp. DSM 115977 | *Micromonospora vinacea* DSM 101695^T^ | FN658651 | 99.06 |
| Strain sp. DSM 115977 | *Micromonospora salmantinae* PSH03^T^ | MN658728 | 99.30 |
| Strain sp. DSM 115977 | *Micromonospora alfalfae* MED01^T^ | MN658725 | 99.30 |
| Strain sp. DSM 115977 | *Micromonospora brunena* DSM 43814^T^ | X92605 | 99.09 |
| Strain sp. DSM 115977 | *Micromonospora profundi* DSM 45981^T^ | KF494813 | 99.31 |
| Strain sp. DSM 115977 | *Asanoa ferruginea* DSM 44099^T^ | X93199 | 98.17 |

**Table S2.** Antimicrobial bioassay of strain DSM 115977^T^ against Gram negative and Gram-positive pathogenic bacteria and yeast after 3 days of incubation of the inoculated plates. The test was performed in duplicates.

|  | **Inhibition zone (mm)** | |
| --- | --- | --- |
| **Reference strains** | **Medium NL800** | **Medium R5** |
| *E. coli* *ΔtolC* JW5503-1 | 10/13 | 0 |
| *Proteus vulgaris* DSM 2140 | 0 | 10 |
| *Staphylococcus aureus* DSM 18827 | 10/12 | 0 |
| *E. faecium* DSM 20477 | 0 | 0 |
| *Candida albicans* DSM 1386 | 0 | 0 |

**Table S3**. BGCs associated with specialised secondary metabolites of strain DSM 115977^T^ and *M. echinofusca* DSM 43913^T^ using AntiSMASH webserver.

| **Type** | **Most similar known cluster** | **DSM 115977**^T^ | **DSM 43913**^T^ |
| --- | --- | --- | --- |
| Hydrogen-cyanide | Aborycin | - | 14% |
| Lanthipeptide-class-ii | Actinoallolide A | - | 20% |
| Lanthipeptide-class-iii | SapB | 100% | 100% |
| Lanthipeptide-class-iv | Labyrinthopeptin A2/labyrinthopeptin A1/labyrinthopeptin A3 | - | 40% |
| NRPS | Azicemicin B | 13% | 13% |
| NRPS | Bengamide | 16% | - |
| NRPS | Lipopeptide 8D1-1/lipopeptide 8D1-2 | 9% | - |
| NRPS,betalactone, lanthipeptide-class-II | Chitinimide B/chitinimide F/chitinimide D/chitinimide A/chitinimide C/chitinimide E/chitinimide G | 58% | 58% |
| NRPS,NRPS-like | Q6402a | - | 5% |
| NRPS,other,betalactone | Melithiazol A | 10% | - |
| NRPS,T1PKS,LAP | Hygrocin A/hygrocin B | 19% | 19% |
| NRPS-like | Livipeptin | 100% | - |
| NRPS-like | Nenestatin | - | 3% |
| NRPS-like, NRPS | Cda1b/cda2a/cda2b/cda3a/cda3b/cda4a/cda4b | 7% | - |
| NRPS-like, T1PKS | Clipibicyclene/azabicyclene B/azabicyclene C/azabicyclene D | 16% | - |
| NRPS-like, T1PKS, NRP-metallophore, NRPS | Kedarcidin | - | 23% |
| Other,betalactone, NRPS | Myxothiazol | - | 28% |
| Ranthipeptide, NRPS-like | Meilingmycin | - | 3% |
| RIPP-like | Lymphostin/neolymphostinol B/lymphostinol/neolymphostin b | 30% | 30% |
| RRE-containing, thiopeptide, LAP | Lactazole | 22% | - |
| T1PKS | Quinolidomicin A | 47% | 71% |
| T1PKS | Sporolide A/sporolide B | 34% | 31% |
| T1PKS | Amycolamycin A/amycolamycin B | - | 11% |
| T1PKS, NRPS | Actagardine | 9% | - |
| T1PKS, NRPS | Crochelin A | - | 12% |
| T1PKS, NRPS | Bleomycin A2/bleomycin B2 | - | 4% |
| T2PKS | Paramagnetoquinone 1/paramagnetoquinone 2 | 25% | - |
| T2PKS | Pradimicin-A | - | 25% |
| T3PKS, thiopeptide, LAP | Loseolamycin A1/loseolamycin A2 | 60% | 80% |
| Terpene | Tetrachlorizine | 13% | 13% |
| Terpene | Meridamycin | 13% | - |
| Terpene | Phosphonoglycans | 3% | 3% |
| Terpene | Isorenieratene | - | 25% |
| Terpene,hydrogen-cyanide | Accramycin A | 5% | - |
| Thioamide-NRP | Anthelvencin A/anthelvencin B/anthelvencin C | - | - |
| Thiopeptide | Dynemicin A | 5% | - |
